# Anatomical Inside-Out Remnant-Preserving Anterior Cruciate Ligament Reconstruction: A Surgical Technique for Biological Anterior Cruciate Ligament Reconstruction

**DOI:** 10.1016/j.eats.2025.103503

**Published:** 2025-03-03

**Authors:** Ali Alayane, Dany Mouarbes, Nicolas Vari, Thomas Ripoll, Corentin Philippe, Maxime Teulieres, Vincent Marot, Etienne Cavaignac

**Affiliations:** a Clinique Universitaire du Sport, Centre Hospitalier Universitaire de Toulouse (CHU), Toulouse, France; b Centre Hospitalier de Perpignan (CHP), Perpignan, France

## Abstract

Preservation of anterior cruciate ligament (ACL) remnants during ACL reconstruction is highly recommended, as it enhances graft healing and contributes to superior functional outcomes. Additionally, preserving ACL remnants is associated with a decreased risk of graft re-rupture. In this Technical Note, we describe an anatomical inside-out ACL reconstruction technique focusing on the optimal preservation of the ACL remnant tissues.

Anterior cruciate ligament (ACL) is the most injured ligament in the knee joint.^1^ Anterior cruciate ligament reconstruction (ACLR) remains the gold standard procedure for achieving anteroposterior knee joint stability. Optimal ACLR depends on several factors, including accurate anatomic graft placement with secure fixation, efficient graft incorporation, revascularization, and ligamentization.^2^ Residual ACL remnants, which include mechanoreceptors and nerve endings, are crucial for promoting graft revascularization and reinnervation.^3^ In addition, the preservation of ACL remnant tissues reduces the incidence of graft rupture after primary reconstruction.^4^^,^^5^

The ACL tibial remnants frequently are debrided to improve the visualization and simplify the surgical procedure.^6^ The main complications of remnant tissue preservation are cyclops lesions, graft impingement within the notch, and loss of extension.^7^ Multiple surgical techniques have been described in the literature for ACL stump preservation,^8^, ^9^, ^10^ with the goal of reconstructing the ACL and properly tensioning the remaining stump to avoid these complications. However, tensioning of the remnants may damage the remaining viable cells and reduce the effectiveness of ACL stump preservation.

In this Technical Note, we describe an arthroscopic technique for anatomical and biological inside-out ACLR aiming to preserve ACL remnants and synovial tissues (Video 1). This technique focuses on the tips and tricks of the tibial tunnel creation to obtain an accurate positioning and to preserve the ACL remnant tissues viability. Surgical pearls and advantages and disadvantages are described in Table 1.

**Table 1:** Pearls, Advantages and Disadvantages for Anatomical Inside-Out Remnant-Preserving ACL Reconstruction, an Optimal Surgical Technique for Biological ACL Reconstruction

Pearls	• The shaver should be passed beneath the attached anteromedial fibers of the ACL remnants, and cleaning of the femoral notch should be performed in a posterior-to-anterior direction, with the shaver’s cutting edge kept flush to the femoral condyle. • The guidewire should be stopped when it reaches the subchondral bone, then its overdrilled using the “knock-on-the-door” technique to allow for smooth cartilage passage. Low-speed reaming should be performed within the ACL stump to avoid damaging remnant tissues. • The shaver should be gently passed through the ACL stump in the same direction as the fibers until it reaches the tip, creating an opening while maintaining tension on the stump. • Final knee extension is performed to ensure the absence of soft-tissue interposition between the graft and the notch.
**Advantages**	• Preservation of ACL stump mechanoreceptors and vascularity enhance graft healing and ligamentization. • No need for remnant tensioning stitch. • Improved knee stability and better functional outcome. • Lower graft re-rupture rate.
Disadvantages	• Minimal risk of cyclops lesion. • Not feasible in cases with poor-quality remnant tissues. • Not feasible in revision cases. • Risk of ACL graft interposition during graft passage • Requires a moderate learning curve to achieve an optimal preservation of ACL remnants.

ACL, anterior cruciate ligament.

## Surgical Technique

### Patient Setup

Under general anesthesia, the patient is placed in supine position with a tourniquet applied on the operative thigh. The knee is then evaluated for full range of motion and confirmed to be stable on the foot roll at 90° of flexion.

The Lachman test is performed preoperatively to confirm anterior knee joint instability, whereas the pivot shift test is conducted to assess posterolateral knee instability.

### Standard Anteromedial and Anterolateral Portals Creation

**Fig 1:**
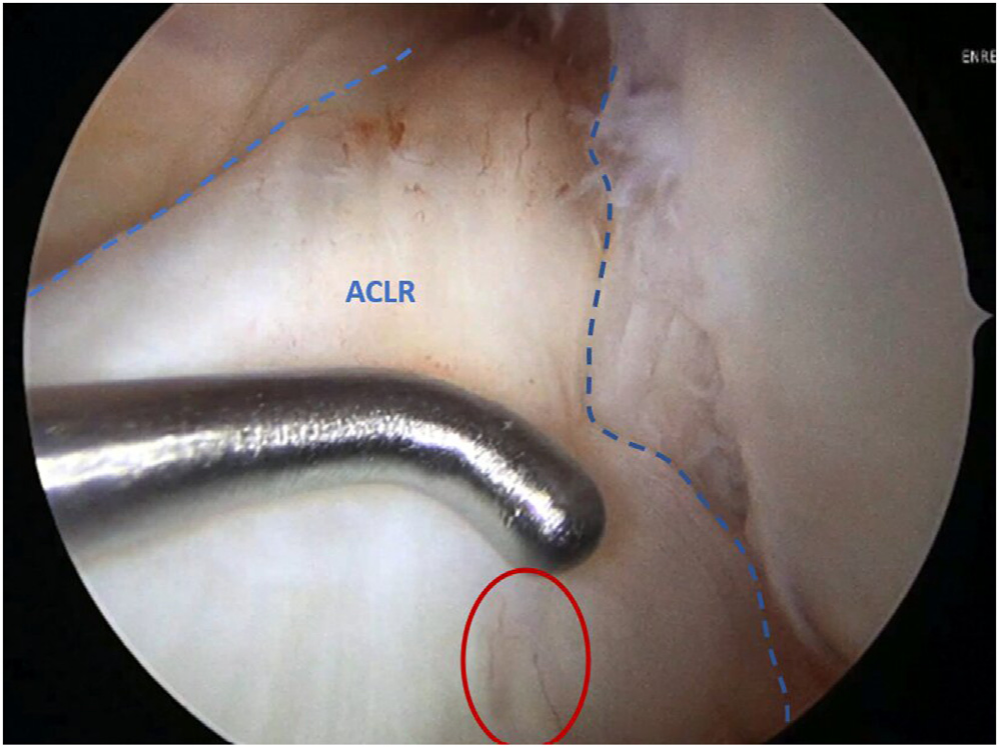
Arthroscopic view of the left knee at 90° of flexion, with the arthroscope in the anterolateral (AL) portal, assessing the quality and vascularity (red circle) of the anterior cruciate ligament remnant (ACLR) tissue using a probe.

Standard anteromedial (AM) and anterolateral portals are established. Using a 30° arthroscope, the length of the ACL remnants and their femoral attachment are assessed, and the vascularity of the remnant tissue is confirmed (Fig 1).

### Femoral Notch Preparation

Femoral notch debridement is meticulously performed using a shaver, ensuring the preservation of the femoral attachment of the anterior fibers of the ACL remnant tissues. Cleaning of the femoral footprint is performed in a posterior-to-anterior and proximal-to-distal direction until the femoral notch outlet is clearly exposed.

### Tibial Tunnel Creation

**Fig 2:**
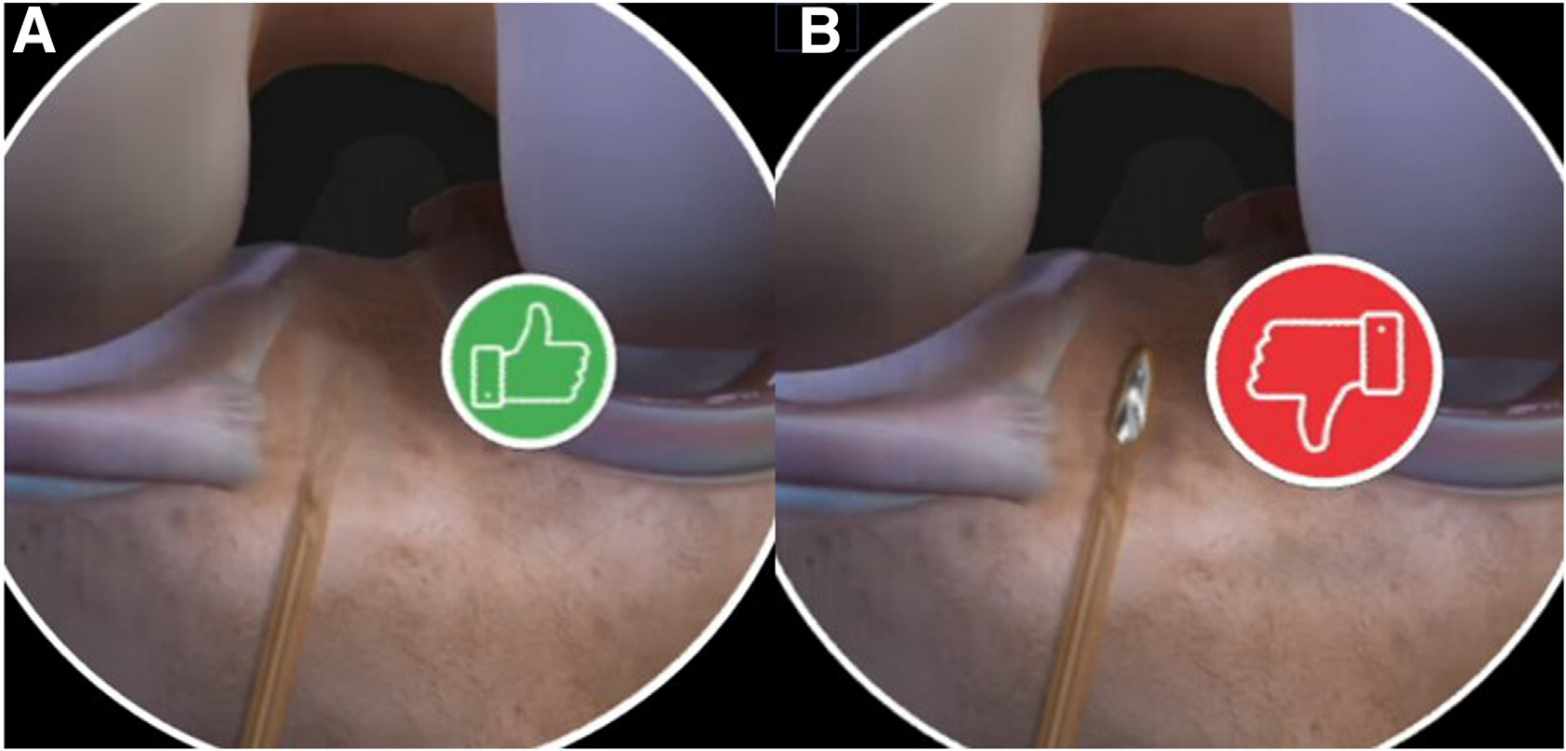
A schematic illustration highlighting the importance of maintaining the guidewire within the subchondral bone of the tibial plateau during tibial tunnel preparation.

The ring of the tibial guide is positioned at the center of the ACL remnants. A guidewire is then inserted slowly through the anterior tibial cortex, just above the hamstrings tendon insertion until it reaches the subchondral bone of the tibial plateau (Fig 2).

The guidewire is initially overdrilled using a 6-mm diameter reamer with the “knock-on-the door” technique. This technique involves tapping the subchondral bone with a slow-speed drill to feel the subchondral cortex and stopping the drill immediately after crossing the second cortical bone. A slow rotation speed is used to complete the drilling of the intra-articular part of the tibial tunnel, minimizing damage to the stump as described by the SAMBBA technique.^8^ The guidewire is then repositioned within the ACL remnant tissues and over-drilled with a 9-mm diameter drill bit, using the same maneuver to preserve the intra-articular ACL remnant tissues and vascularity.

**Fig 3:**
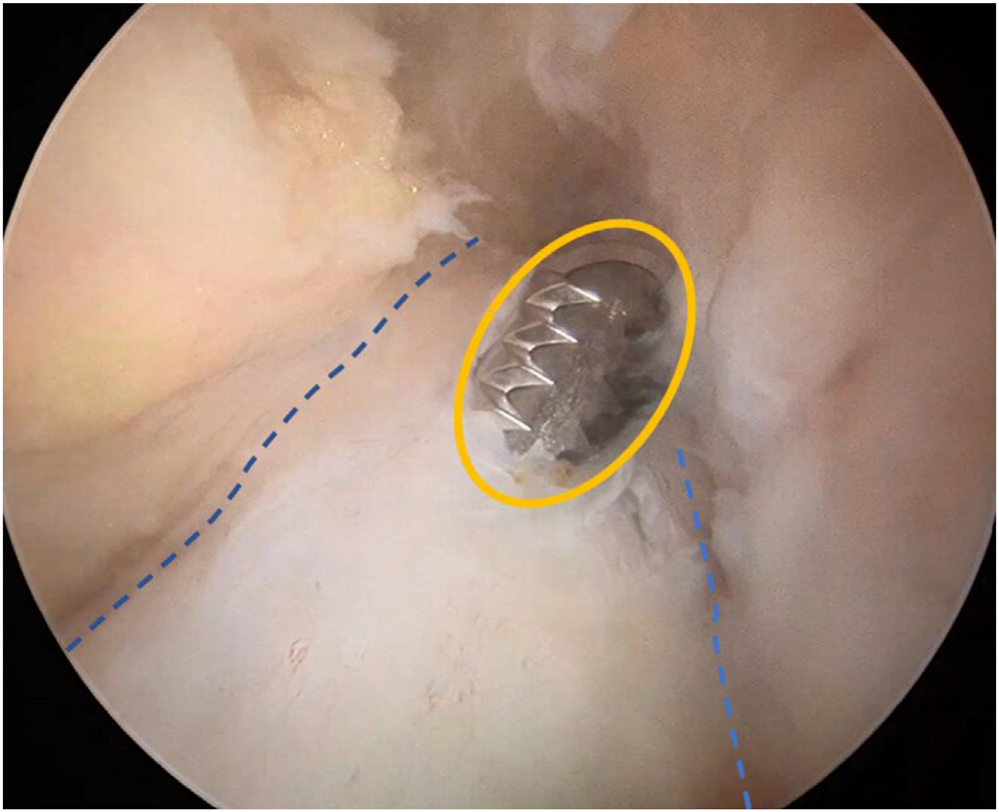
Arthroscopic view of the left knee at 90° of flexion, with the arthroscope inserted through the anterolateral portal, showing the creation of the ACL stump opening (highlighted in yellow) using a shaver to allow passage of the ACL graft. (ACL, anterior cruciate ligament.)

A shaver is inserted through the tibial tunnel to clean the bone debris and it’s advanced carefully along the alignment of the ACL remnant fibers until it reaches the tip. A small proximal opening is then made to enable the passage of the graft later (Fig 3).

### Femoral Tunnel Creation

A guidewire is introduced through the femoral inside-out guide (4-mm eyelet pin) and positioned at the prepared femoral footprint within the knee joint at 90° of flexion. The knee is then flexed to 120°, and the guidewire is inserted into the femur until it exits the skin over the lateral femoral condyle. The reamer is carefully introduced over the guidewire, contacting the bone before drilling a 20-mm socket tunnel. A loop suture is passed though the femoral tunnel and retrieved form the tibial tunnel. Using a shaver through the AM portal, intra-articular debris are removed to reduce the risk of postoperative knee joint inflammation.

### Graft Length Measurement and Preparation

**Fig 4:**
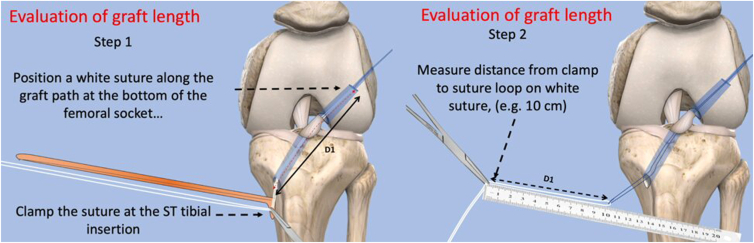
A schematic illustration of the preparation of the pediculated anterior cruciate ligament semitendinosus (ST) graft. The distance (D1) from the clamp to the end of the white suture at the base of the femoral socket represents the required graft length from the ST insertion to the bottom of the femoral socket.

**Fig 5:**
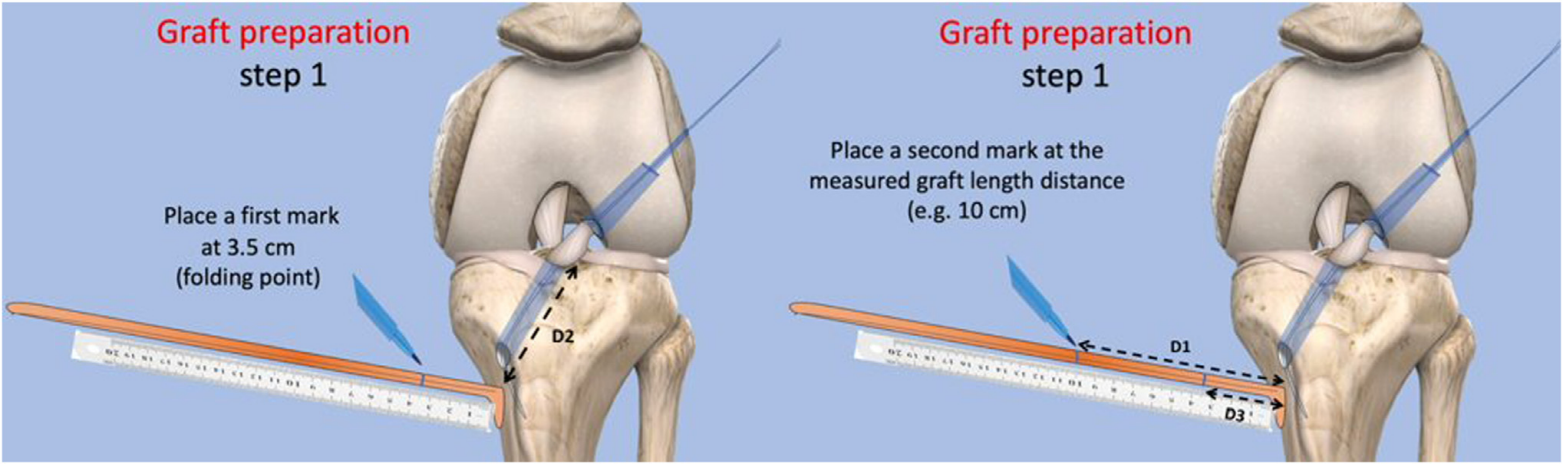
A schematic illustration of the anterior cruciate ligament semitendinosus (ST) graft preparation. The distance (D1) from the clamp to the end of the white suture at the base of the femoral socket represents the required graft length from the ST insertion to the bottom of the femoral socket. The distance (D2) represents the length from the intra-articular entry point of the tibial tunnel to the ST tendon insertion. A first mark is made on the ST tendon at a distance D3 = D2 − 2 cm, with the 2 cm representing the tripled part of the graft intended to be within tibial tunnel/ A second mark is made at D1 from the ST tendon insertion.

Accurate measurement of graft length is essential to achieve appropriate tensioning from the tibial insertion to the bottom of the femoral tunnel. A white suture is passed through the tibial tunnel using the loop suture until it reaches the bottom of the femoral socket under direct arthroscopic visualization through the AM portal. A clamp is then placed on the white suture at the level of the semitendinosus (ST) tendon insertion. The distance (D1) from the clamp to the end of the white suture at the bottom of the femoral socket represents the required graft length from the ST insertion to the bottom of the femoral socket (Fig 4). A depth gauge is used to measure the distance (D2) form the intra-articular entry point of the tibial tunnel to the insertion of the ST tendon (Fig 5).

The ST tendon, which is harvested at the beginning of the procedure and kept in a vancomycin-soaked compress to prevent infection, remains attached at its tibial insertion. A first mark is made on the ST tendon at a distance D3 = D2 − 2 cm, with the 2 cm representing the tripled part of the graft intended to be within tibial tunnel. A second mark is made at D1 from the ST tendon insertion (Fig 5).

**Fig 6:**
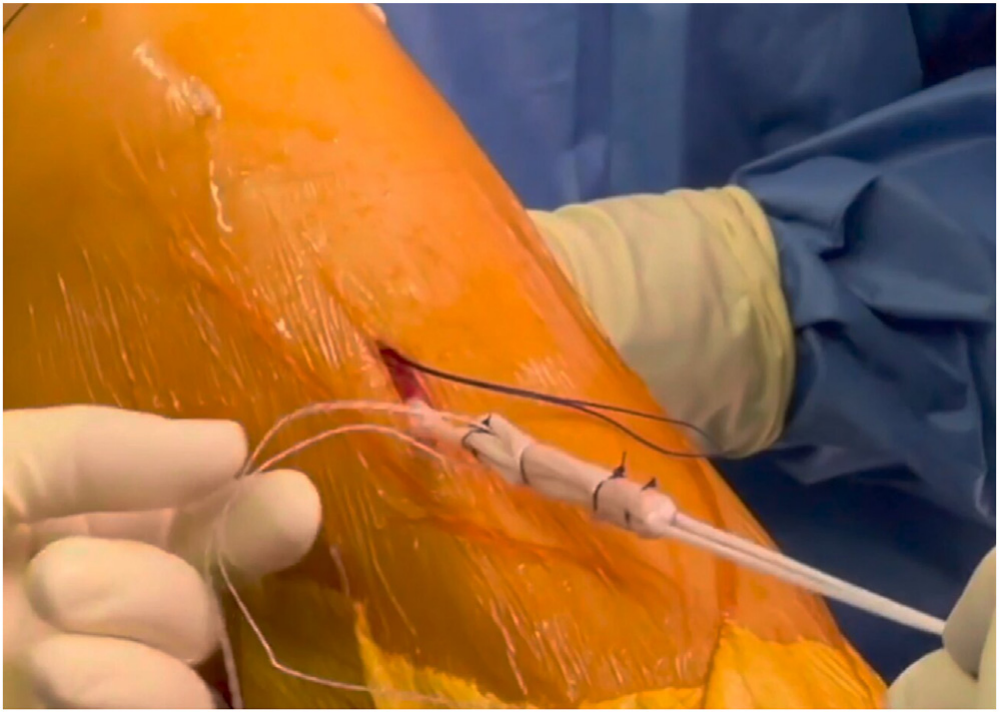
Intraoperative image showing the prepared tripled semitendinosus anterior cruciate ligament graft. The semitendinosus tendon attachment to the tibial insertion is preserved to enhance graft healing.

A tripled or quadrupled ST tendon graft is folded and sutured to itself between both marks on the basis of the thickness of the ST tendon and looped with an adjustable TightRope RT implant (Arthrex) (Fig 6).

### Graft Passage and Fixation

**Fig 7:**
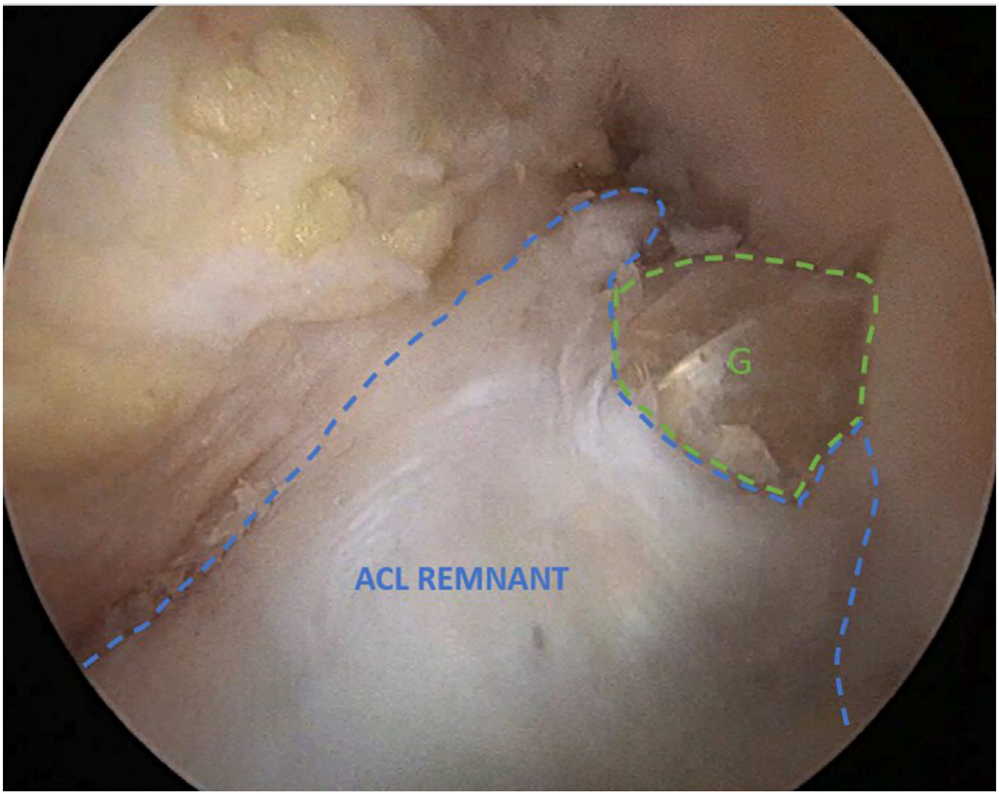
Arthroscopic view of the left knee at 90° of flexion, with the arthroscope inserted through the anterolateral portal, showing the semitendinosus anterior cruciate ligament (ACL) graft positioned within the preserved ACL remnant tissue at the end of the procedure. Full knee extension is performed to confirm the absence of soft-tissue interposition. (G, graft.)

While maintaining the knee at 90° of flexion, the prepared ACL graft is passed through the tibial tunnel using the loop suture, and the endobutton is pulled into the femoral tunnel under direct arthroscopic control via the AM portal and flipped on the lateral femoral condyle. The ACL graft is then gradually pulled up to fill the femoral tunnel under arthroscopic visualization through the anterolateral portal. Adequate graft tension is checked using a probe and final arthroscopic inspection of the graft within the preserved vascularized ACL stump is performed (Fig 7). knee extension is performed to ensure the absence of soft-tissue interposition between the graft and the notch. Tibial graft fixation is completed using an interference screw of the same diameter as the tibial tunnel while maintaining the knee in a reduced position.

### Postoperative Protocol

The postoperative protocol includes immediate weight-bearing using crutches and early progressive range-of-motion exercises to restore full knee extension and enhance quadriceps muscle contraction. The patient is allowed to return to sports, starting with nonpivoting activities at 6 months, followed by pivoting sports 9 months postoperatively.

## Discussion

The potential role of ACL remnants has been extensively researched, with mechanoreceptors in the stump affect motor function.^11^ Preserving these remnants acts as a biological sleeve, enhancing graft revascularization and ligamentization, while also maintaining proprioceptive function.^12^ In addition, remnant-preserving ACLR shows similar tibial tunnel accuracy to traditional techniques that remove the stump for better landmark visibility.^13^

Numerous surgical techniques for ACLR with remnant preservation have been described in the literature.^14^, ^15^, ^16^ However, full preservation of the ACL stump has not always been achieved as intended. In addition, placing a suture on the ACL stump to apply tension may compromise the vascularity of the remnant tissue, potentially diminishing the benefits of stump preservation.

Our Technical Note aims to simplify ACLR with stump preservation by using the standard inside-out technique. After checking the ACL stump vascularity, the debridement of the femoral footprint is performed from posterior to anterior to avoid remnant injury. Subsequently, during tibial tunnel creation, the “knock-on-the-door” technique aims to smoothly pass through the cartilage, thereby preventing damage to the remnant tissues and avoiding intra-articular osteochondral fractures of the tibial eminence. In addition, advancing the shaver in the proper direction within the stump to create the tip opening under tension, thereby preventing the need for a stump tension suture.

The main risk associated with this technique is cyclops lesion formation resulting in postoperative extension deficit. This complication can be prevented by assessing knee joint extension at the end of the procedure to confirm the absence of soft-tissue interposition.

In our perspective, we believe that our technique offers a simplified approach for ACLR with optimal remnant preservation. The authors encourage the “knock-on-the-door” technique to protect the ACL stump during tibial tunnel creation. In addition, keeping the graft attached to the tibia is also recommended to improve knee joint stability and decrease graft rupture rate.^17^

## Disclosures

The authors declare the following financial interests/personal relationships which may be considered as potential competing interests: E.C. reports a relationship with Arthrex that includes consulting or advisory. All other authors (A.A., D.M., N.V., T.R., C.P., M.T., V.M.) declare that they have no known competing financial interests or personal relationships that could have appeared to influence the work reported in this paper.

## Supplementary Data

Video 1 This instructional video demonstrates an anatomical, inside-out remnant-preserving anterior cruciate ligament (ACL) reconstruction. The first stage involves evaluating the ACL remnant tissue to assess the feasibility of performing this technique. The second stage consists of femoral notch debridement from the posterior to anterior to expose the ACL femoral footprint. Subsequently, tibial tunnel creation is performed with a meticulous 2-step reaming process to allow passage of the semitendinosus ACL graft through the preserved stump. The goal of this procedure is to achieve a biological ACL reconstruction with optimal ACL remnant preservation, avoiding the need for a tension suture on the ACL stump, thus maintaining vascularity and enhancing ACL graft ligamentization.
